# Gender disparity and temporal trend of liver cancer in China from 1990 to 2019 and predictions in a 25-year period

**DOI:** 10.3389/fpubh.2022.956712

**Published:** 2022-08-26

**Authors:** Tingting Yue, Ming Xu, Ting Cai, Haizhen Zhu, Mahmoud Reza Pourkarim, Erik De Clercq, Guangdi Li

**Affiliations:** ^1^Hunan Provincial Key Laboratory of Clinical Epidemiology, Xiangya School of Public Health, Central South University, Changsha, China; ^2^Institute of Pathogen Biology and Immunology of College of Biology, Hunan Provincial Key Laboratory of Medical Virology, State Key Laboratory of Chemo/Biosensing and Chemometrics, Hunan University, Changsha, China; ^3^Department of Microbiology, Immunology and Transplantation, Rega Institute for Medical Research, KU Leuven, Leuven, Belgium; ^4^Health Policy Research Center, Institute of Health, Shiraz University of Medical Sciences, Shiraz, Iran; ^5^Blood Transfusion Research Center, High Institute for Research and Education in Transfusion Medicine, Tehran, Iran; ^6^Hunan Children's Hospital, Changsha, China

**Keywords:** liver cancer, gender disparity, temporal trend, prediction, alcohol use, tobacco use

## Abstract

**Objective:**

This study aims to reveal epidemiological features and trends of liver cancer (LC) in China.

**Methods:**

We retrieved data from the Global Burden of Disease database 2019. Joinpoint regression was used to examine the temporal trend of LC. Future trends of LC were estimated using the Nordpred.

**Results:**

The incidence, mortality, and disability-standardized life year (DALY) rate of LC declined in China from 1990 to 2019. Among >210,000 LC cases in 2019, the LC incidences were nearly 3.15 times higher in males than in females. LC cases and LC-associated deaths were mostly found among patients aged 65 to 69 years. The proportion of LC attributable to hepatitis B decreased over time, whereas the proportions of LC attributable to hepatitis C, alcohol use, and non-alcoholic steatohepatitis increased modestly from 1990 to 2019. The majority of LC-associated deaths could be traced to four risk factors: smoking (20%), drug use (13.6%), alcohol use (11.7%), and high body mass index (10.1%). Based on the Nordpred prediction, there will be a steady decline in the incidence (39.0%) and mortality (38.3%) of liver cancer over a 25-year period from 2020 to 2044.

**Conclusion:**

The disease burden of liver cancer in China has declined over the past 30 years. However, it remains important to control liver cancer among high-risk populations, especially elderly males with obesity, alcohol use, tobacco use, and/or drug abuse.

## Introduction

Primary liver cancer (LC), the sixth most common cancer worldwide (1), is known for its insidious onset, complex etiology, extraordinarily heterogeneous, high degree of malignancy, and high recurrence and metastasis (2, 3). In 2019, a total of >534,000 new LC cases and >484,000 LC-associated deaths were estimated (4). By 2050, liver cancer probably affects more than 1 million patients annually (5). Liver cancer is associated with many risk factors, including chronic HBV or HCV infection, alcoholism, diabetes, non-alcoholic steatohepatitis, cirrhosis, aflatoxin, obesity, and tobacco use (6).

In China, LC is the second leading cause of cancer deaths, and the first leading cause is lung cancer (7). From 1990 to 2019, LC incidence and mortality declined in China (8), but the disease burden remains high due to the large population in China. In 2019, approximately 39.4% of global LC cases occurred in China (9). At least 60% of liver cancer is caused by HBV or HCV infection (10). China has implemented a series of strategies to control the spread of HBV and HCV, for instance, by introducing HBV vaccination in children (11, 12), blood transfusion screening of hepatitis viruses, and prevention of mother-to-child transmission (13, 14). Despite these enormous efforts, the absolute number of HBV- or HCV-infected patients remains high in China. According to the Global Burden of Disease (GBD) 2019, more than 23 million HBV infections occurred in China in 2019, and 0.6 million for HCV infections. Due to the dynamics of socioeconomic, dietary, lifestyle and living conditions, there is a growing concern for the impact of other LC-associated risk factors such as obesity, smoking, alcohol use, and non-alcoholic steatohepatitis (8, 15–17). Therefore, it remains important to evaluate the epidemic burden of LC from current and future perspectives.

Because China has the largest number of LC cases among all countries (8), this study aims to evaluate the disease burden of LC in China and estimate the future trend of liver cancer from 2019 to 2044 based on a multinational collaborative project from the GBD2019 (18, 19). This study will report epidemiological features of liver cancer and shed light on the management of liver cancer in China.

## Methods

### Data sources

Data used in this study was extracted from the Global Burden of Disease 2019 (GBD2019) database (https://ghdx.healthdata.org/gbd-2019), which provides a comprehensive epidemiological database for 369 diseases and injuries (18, 19). We obtained complete data on the incidence, mortality, disability-standardized life year (DALY) rates, and risk factors of liver cancer by gender in China from 1990 to 2019 from GBD2019. The GBD2019 database included data from 204 countries and territories based on a variety of sources such as national surveys, censuses, vital statistics, and other health-related data sources. The data from these sources are used to estimate the incidences, mortality, and attributable risk based on the estimation method of the Bayesian meta-regression model DisMod-MR 2.1 (20). The LC data from China were mainly collected from the surveillance data of the China Disease Surveillance Point System and the registration data collected by the Chinese Center for Disease Control and Prevention (19, 20). The proportions of the five etiologies (HBV, HCV, alcohol use, non-alcoholic steatosis hepatitis, and other causes such as fluke and aflatoxin) from meta-analyses were used as inputs for the DisMod-MR 2.1 model which estimates the incidences, mortality, and proportions of liver cancer in the context of different etiologies (21). The GBD2019 estimation of attributable burden followed the general framework, the so-called Comparative Risk Assessment (CRA), which had been established for risk factors assessment (22). The protocol of the CRA method can be briefly summarized as follows. (i) The method includes risk-outcome pairs that meet the criteria with convincing evidence. (ii) It collects information on the relative risk by the level of exposure or by the cause of mortality/morbidity from pooled cohorts, meta-analyses of cohorts, and case-control data. Meta-analyses are used to estimate relative risks of mortality/morbidity for risk-outcome pairs. (iii) It uses DisMod-MR 2.1, spatiotemporal Gaussian process regression, or alternative methods to estimate the exposure levels of each age-sex-region-year. (iv) It collects theoretical minimum exposure levels from published trials or cohorts. (v) It calculates population attributable fractions and attributable burden. (vi) It estimates the disease burden attributable to various combinations of risk factors such as high body mass index (BMI) and high fasting blood glucose (22, 23).

### Definitions

A high body-mass index (BMI) was defined as a value above 25 kg/m^2^. The category of tobacco use included smoking, second-hand smoke, and smokeless tobacco use (e.g., chewing tobacco). Smoking was defined as the current/former use of any smoked tobacco product (e.g., cigarettes, pipes, cigars, shisha, bidis, kreteks, and/or other local tobacco products) on a daily or occasional basis (24, 25). Secondhand smoke was defined for those non-smokers who lived with current daily smokers and had the average daily exposure to indoor air particulate matter (with an aerodynamic diameter <2.5μm) from second-hand smoke (26). Smokeless tobacco use was defined as the current use of any smokeless tobacco product (27). Alcohol use was defined as the average pure alcohol consumption ≥10 g/day in current drinkers who had consumed alcohol during the past 12 months (28). The consumption of pure alcohol (males: ≥60 g, females: ≥48 g) on a single occasion was defined as binge drinking (27). Drug use was defined as the regular use of opioids, cannabis, cocaine, amphetamines, or ever-injected drugs (26, 27).

### Statistical analysis

To quantify the disease burden of LC in China, we used the age-standardized incidence rate (ASIR), age-standardized DALY rate (ASDR), and age-standardized mortality rate (ASMR) that take into account the age structure differences, as described previously (29). Joinpoint regression models (https://surveillance.cancer.gov/joinpoint/) were used to calculate annual percentage changes (APCs) and average annual percentage changes (average APCs). The grid search method and permutation tests were used to determine the joinpoint model and the optimal model, respectively.

We used the age-period-cohort model and the power-link function in the R Nordpred package to project the future trend of incidence and mortality of LC in China, taking into account the impact of population structure. As an R package, Nordpred was built based on the age-period-cohort model, a well-established estimation method for cancer incidence and/or mortality prediction (30, 31). The measured variable of incidence or mortality was modeled using input variables such as age, calendar period, and birth cohort. The model can be briefly expressed as $R_{\alpha p}={\left(A_{\alpha }+D\cdot p+P_{p}+C_{c}\right)}^{5}$, where *R*_α*p*_ indicates the incidence or mortality numbers from the age group α during the period p; *A*_α_ indicates the age component for the age group α; D represents the common drift parameter that summarizes the linear component of the trend; *P*_*p*_ denotes a non-linear period component during the period p; and *C*_*c*_ indicates the non-linear cohort component of the cohort c (32). The performance of Nordpred-based predictions has been validated and optimized by many studies (33–35). In our study, we analyzed the incidence and mortality data of liver cancer in China for a five-year period (1990 to 1994, 1995 to 1999, …, 2015 to 2019) and the 5 years old age groups except those under 14 years old (<15y, 15y to 19y, 20y to 24y, … ≥95y). The prediction of liver-cancer incidence/mortality was conducted in the 5-year period (2020 to 2024, 2025 to 2029, 2030 to 2034, 2035 to 2039, 2040 to 2044). The estimated population of China and standard population structure were collected from the United Nations World Population Prospects 2019 Revision (https://population.un.org/wpp/) and the WHO standard population structure (https://seer.cancer.gov/stdpopulations/world.who.html).

Statistical analysis was performed using R (version 4.1.0), Joinpoint Regression Program (version 4.9.0.0), and GraphPad Prism (version 8.0.1). *P*-value <0.05 was considered statistically significant. All rates are reported per 100 000 person-years.

## Results

### Temporal trend of liver cancer (LC) in China

**Figure 1:**
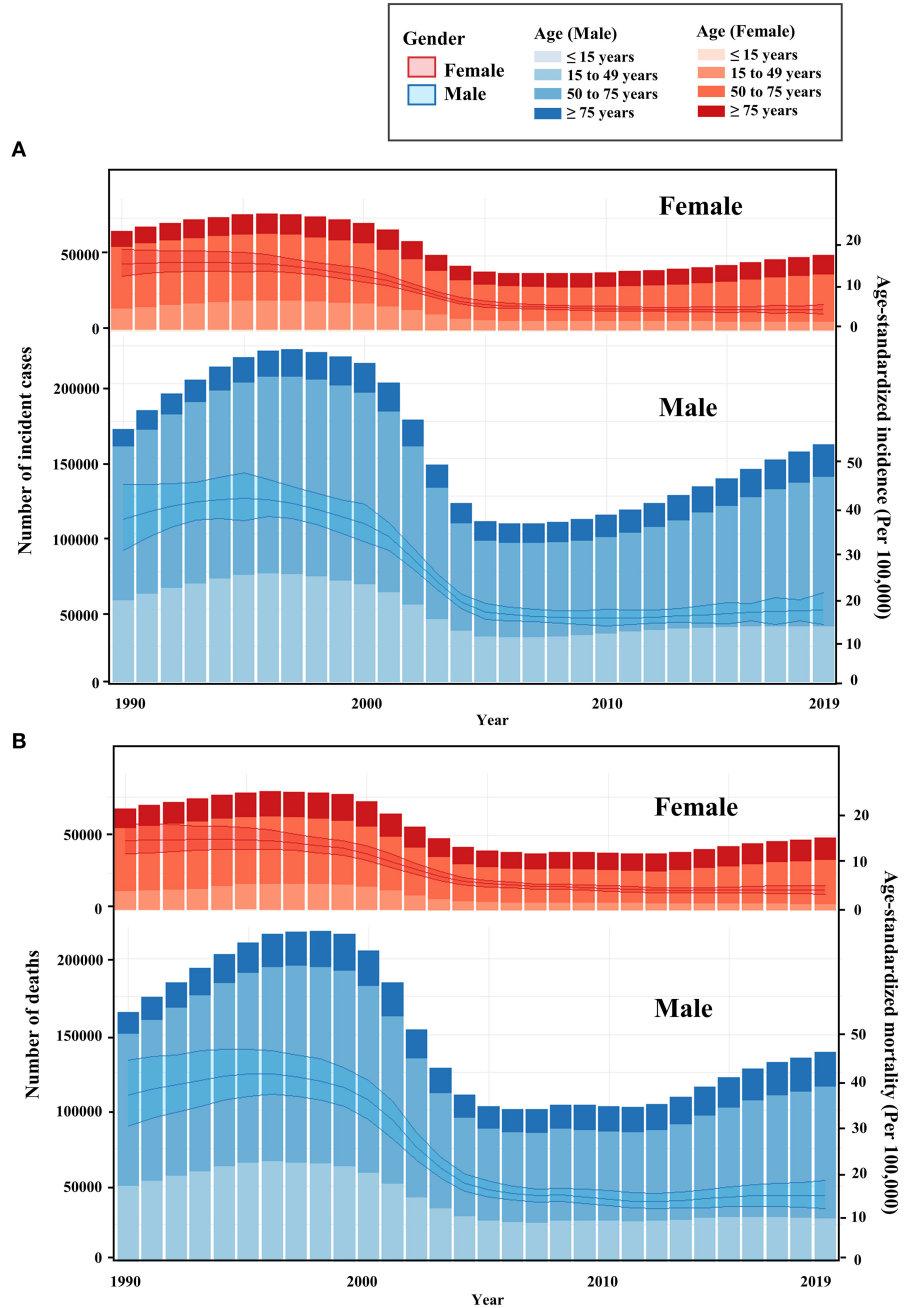
Temporal trend of liver cancer in China. **(A)** Temporal trend of age-standardized incidence and number of cases for liver cancer from 1990 to 2019 in China; **(B)** Temporal trend of age-standardized mortality and number of deaths for liver cancer from 1990 to 2019 in China. The bar was the number of liver cancer cases and liver cancer-associated deaths from 1990 to 2019. The line with 95% UI represents incidence and mortality from 1990 to 2019.

In 2019, approximately 210,462 LC cases and 187,699 LC-associated deaths were reported in China (Table 1). The majority of LC cases were males, and the number of male cases was 3.15 times higher than that of female cases. Compared with the data in 1990, the total number of LC cases and LC-associated deaths in 2019 decreased by 11.1 and 19.3%, respectively (Figure 1). The decrease in females was more dramatic than that in males, with a significant decrease in LC cases (females: 24.0%, males: 6.1%) and LC-associated deaths (females: 27.9%, males: 15.7%). The DALY rate showed a similar downward trend over years (Supplementary Figure S1).

**Table 1:** Incidences, mortality, and DALY rates of liver cancer in China in 1990 and 2019.

	**Patient number**	**Patient number**	**Change (%)**	**ASR in 1990**	**ASR in 2019**	**Change (%)**
	**in 1990**	**in 2019**		**(Per 100,000)**	**(Per 100,000)**	
**Incidence**						
Total	236,825	210,462	−11.1	25.7	10.5	−58.8
Females	66,709	50,675	−24.0	15.0	4.9	−67.3
Males	170,115	159,787	−6.1	36.4	16.4	−54.9
<25 years	5,285	1,395	−73.6	2.4	1.1	−54.2
25 to 49 years	65,258	43,508	−33.3	16.0	7.7	−51.9
50 to 74 years	144,013	130,842	−9.1	83.3	31.4	−62.3
≥75 years	22,269	34,717	55.9	114.8	57.8	−49.7
**Mortality**						
Total	232,449	187,699	−19.3	26.0	9.4	−63.8
Females	67,543	48,674	−27.9	15.6	4.8	−69.2
Males	164,905	139,025	−15.7	36.7	14.6	−60.2
<25 years	4,273	881	−79.4	1.8	0.6	−64.6
25 to 49 years	59,579	33,222	−44.2	14.6	5.9	−59.5
50 to 74 years	141,337	11,542	−18.3	81.7	27.7	−66.1
≥75 years	27,260	38,144	39.9	140.5	63.5	−54.8
**DALY**						
Total	7,577,768	5,325,460	−29.7	769	264	−65.7
Females	1,987,369	1,179,666	−40.6	420	116	−72.4
Males	5,590,399	4,145,795	−25.8	1,103	415	−62.4
<25 years	322,471	65,931	−79.6	129.5	45.9	−64.6
25 to 49 years	2,855,373	1,563,215	−45.3	697.8	277.3	−60.3
50 to 74 years	4,033,010	3,214,174	−20.3	1,233.5	409.1	−66.8
≥75 years	366,914	482,141	31.4	1,339.4	533.5	−60.2

DALY, Disability-adjusted life years; ASR, Age-standardized rate.

The highest number of LC cases and LC-associated deaths were observed among patients aged between 65 and 69 years (Supplementary Figures S2A,S2B). Most LC cases occurred in males aged from 50 to 54 years and females aged from 65 to 69 years (Supplementary Figures S2C,S2E). LC deaths in both males and females mostly occurred in patients aged from 65 to 69 years (Supplementary Figures S2D,S2F). From 1990 to 2019, the overall trends of ASIR and ASMR decreased over time. The ASIR dropped dramatically from 2001 to 2005, with a decreased APC_2001_-_2005_ of −17.30% in males and −15.43% in females (Table 2). The most significant decrease in ASMR occurred between 2000 and 2004, with a decreased APC_2000−2004_ of −17.5% in males and −15.56% in females (Table 2). Since 2012, both ASIR and ASMR in males shared a slightly increasing trend (APC_2010−2019_ of ASIR, 1.6% [1.4 to 1.7%]; APC_2012_-_2019_ of ASMR, 1.6% [1.2 to 2.1%]), whereas the ASIR and ASMR in females remained stable (Table 2).

**Table 2:** Temporal trend of LC incidence and mortality from 1990 to 2019 in China.

**Year**	**All patients**		**Female patients**		**Male patients**	
	**APC (%)**	**Average APC (%)**	**APC (%)**	**Average APC**	**APC (%)**	**Average APC**
	**(95% CI)**	**(95% CI)**	**(95% CI)**	**(%) (95% CI)**	**(95% CI)**	**(%) (95% CI)**
**Age-standardized incidence rate**						
1990 to 1995	1.7 (1.2 to 2.1)	−3.1 (−3.4 to −2.9)	0.4 (−0.3 to 1.0)	−3.8 (−4.1 to −3.5)	2.2 (1.7 to 2.7)	−2.8 (−3.0 to −2.5)
1995 to 1998	−2.5 (−3.8 to −1.2)		−3.2 (−5.1 to −1.3)		−2.2 (−3.6 to −0.8)	
1998 to 2001	−5.8 (−7.1 to −4.4)		−6.6 (−8.4 to −4.7)		−5.3 (−6.9 to −3.8)	
2001 to 2005	−16.9 (−17.4 to −16.3)		−15.4 (−16.1 to −14.7)		−17.3 (−17.8 to −16.8)	
2005 to 2010	−2.0 (−2.5 to −1.6)		−2.6 (−3.0 to −2.2)		−1.5 (−2.0 to −1.0)	
2010 to 2019	1.0 (0.8 to 1.2)		−0.2 (−0.4 to 0.1)		1.6 (1.4 to 1.7)	
**Age–standardized mortality rate**						
1990 to 1996	1.4 (0.9 to 1.9)	−3.4 (−3.7 to −3.1)	0.1 (−0.3 to 0.6)	−4.0 (−4.2 to −3.8)	2.0 (1.4 to 2.6)	−3.1 (−3.4 to −2.7)
1996 to 2000	−3.7 (−4.7 to −2.8)		−4.7 (−5.4 to −3.9)		−3.3 (−4.4 to −2.1)	
2000 to 2004	−17.1 (−18.0 to −16.2)		−15.7(−16.4 to −14.9)		−17.5 (−18.6 to −16.5)	
2004 to 2007	−5.2 (−7.0 to −3.3)		−5.3 (−6.8 to −3.8)		−4.8 (−7.0 to −2.6)	
2007 to 2012	−2.3 (−3.0 to −1.6)		−3.2 (−3.7 to −2.8)		−1.8 (−2.6 to −1.1)	
2012 to 2019	1.2 (0.8 to 1.6)		0.1 (−0.2 to 0.4)		1.6 (1.2 to 2.1)	

APC, Annual percentage change; ASIR, Age-standardized incidence rate; ASMR, Age-standardized mortality rate.

### Risk factors of liver cancer in China

**Figure 2:**
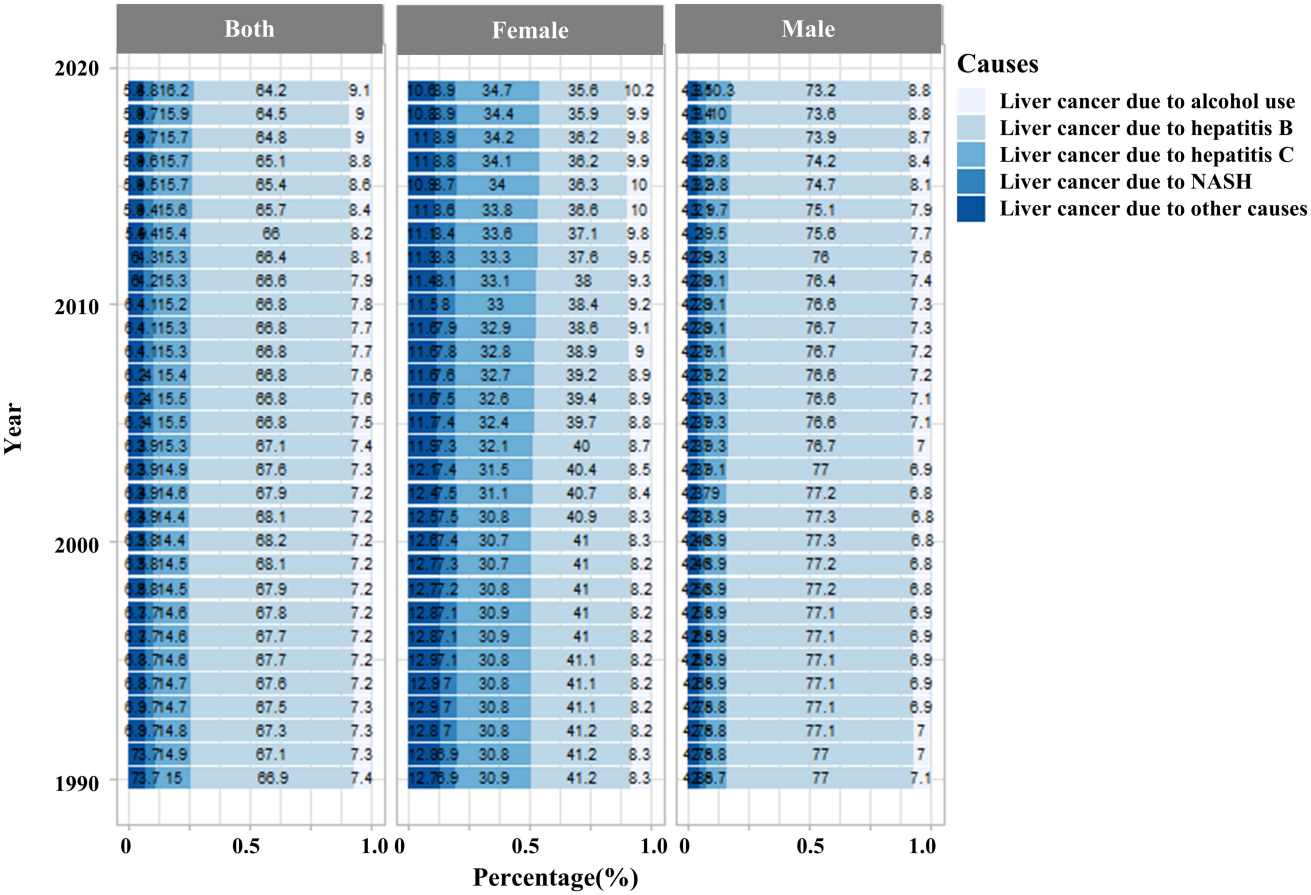
The disease burden of liver cancer incidence rates in the Chinese population is attributable to five risk factors (alcohol use, hepatitis B, hepatitis C, non-alcoholic steatosis hepatitis, and other causes) between 2010 and 2019. (NASH, non-alcoholic steatosis hepatitis).

**Figure 3:**
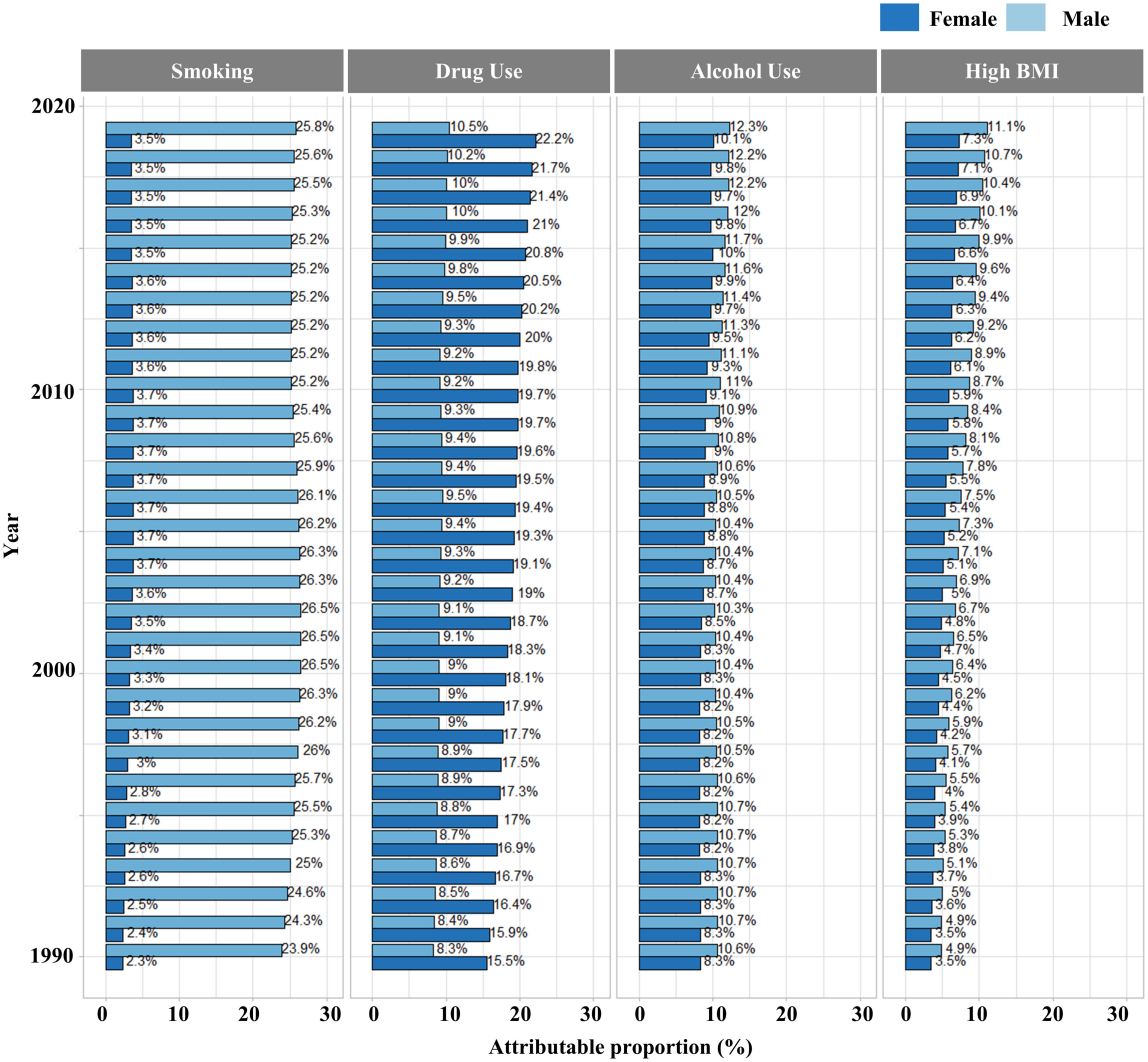
Proportions of LC-associated deaths attributable to four risk factors (alcohol use, drug use, high BMI, smoking) from 1990 to 2019.

We analyzed the proportion of liver cancer caused by five specific etiologies (alcohol use, hepatitis B, hepatitis C, non-alcoholic steatosis hepatitis, and other causes) in Chinese patients with liver cancer. Our results showed that nearly 80% of liver cancer cases in China were attributable to HBV or HCV infection, especially among male patients. HBV/HCV infections remained the leading cause of liver cancer, although the proportion of other causes had increased slightly. Among female patients, the proportion of HBV- and HCV-induced LC was similar, but the proportion of HBV-induced LC was significantly higher than that of HCV-induced LC in male patients (Figure 2). As for the risk factors associated with the deaths, the majority of LC-associated deaths in 2019 can be attributed to smoking (20%), followed by drug use (13.6%), alcohol use (11.7%), and high BMI (10.1%). High BMI also caused a modest increase in LC-associated deaths from 1990 (4.4%) to 2019 (10.1%) (Figure 3).

### Prediction of LC incidence and mortality in China

**Figure 4:**
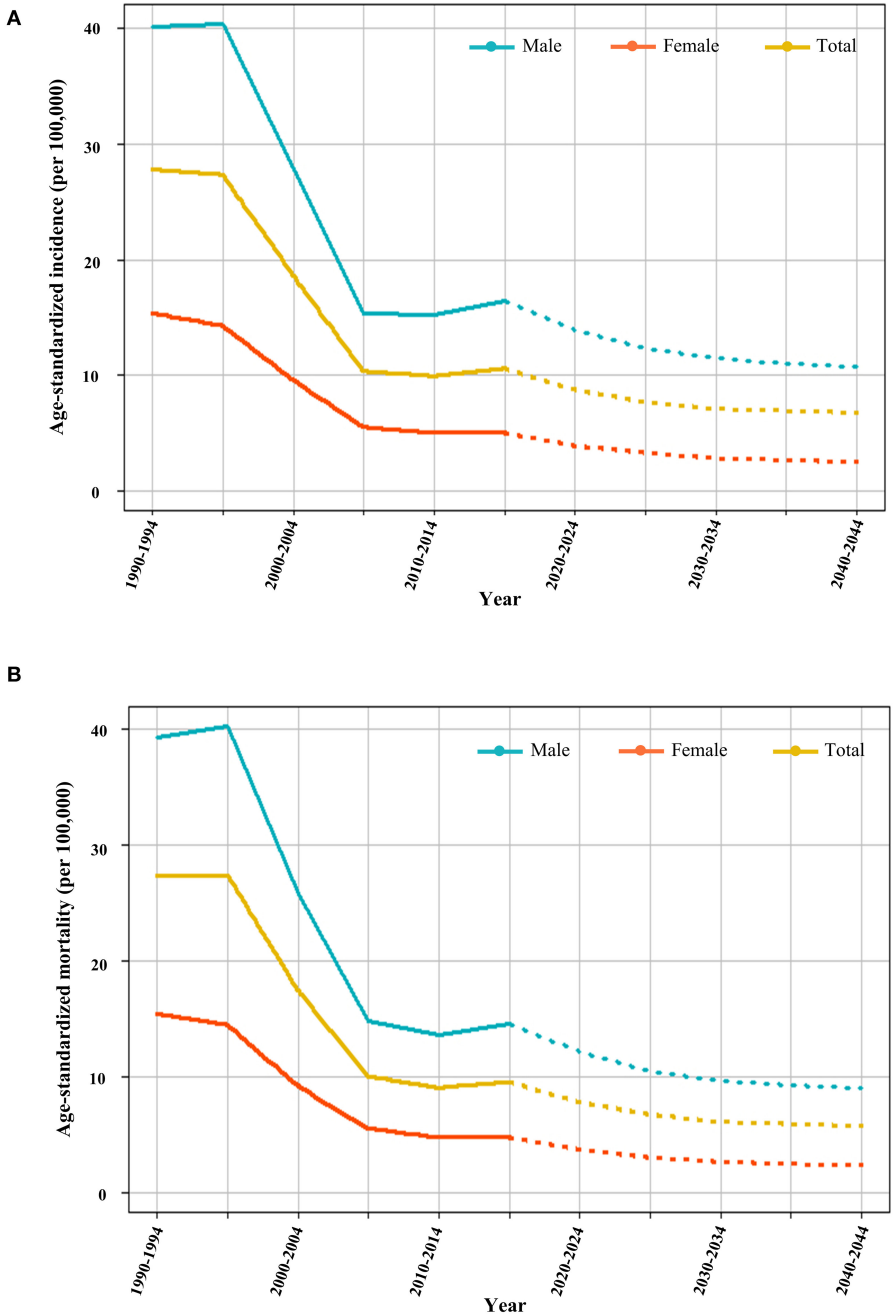
Prediction of incidence and mortality of liver cancer. **(A)** Plots of age-standardized incidence of liver cancer from 1990 to 2044. The solid lines indicate the observed values (1990 to 2019) and the dotted lines are the predicted values (2020 to 2044). **(B)** Plots of LC age-standardized mortality from 1990 to 2044.

Based on the accumulated data from 1990 to 2019, we projected the LC incidence and mortality in China from 2020 to 2044. Based on the prediction from the Nordpred software (see Methods), the age-standardized incidence of LC will decrease steadily from 10.7 (per 100,000 population) in 2019 to 6.9 (per 100,000 population) by 2044. The LC incidence will be still higher in males (10.8 per 100,000) than in females (3.3 per 100,000) in 2044 (Figure 4A). In the evaluation of age-standardized mortality rates, similar findings were found in the prediction of LC incidence (Figure 4B). In 2019, the mortality rate for LC was 9.7 per 100,000, with 14.7 per 100,000 in male patients and 4.87 per 100,000 in female patients. By 2044, the mortality rate for LC is expected to drop to 5.9 per 100,000, 9.0 per 100,000 for men, and 2.4 per 100,000 for women.

## Discussion

The disease burden and risk factors of liver cancer in China have not been fully reported to date. Previous studies only predicted the incidences of liver cancer in China based on the limited data from 1983 to 2007 and from 2000 to 2014 (36, 37). Here, this study reveals the disease burden of liver cancer in China from 1990 to 2019 and the future trend of incidence and mortality from 2020 to 2044. Our study found that the risk of LC incidence and mortality was higher in males than in females. The absolute number of LC incident cases was approximately three times higher in males than in females. Most LC cases were found in males aged between 50 and 54 years, and the average age of male patients was 15 years younger than female patients. This finding is in agreement with a global cancer burden study that liver cancer is one of the most common causes of death in males (38). Based on the GBD data from 1990 to 2019, we observed an increase in LC-associated incident cases, deaths, and DALY rates among patients >75 years old, probably because of the aging population in China (9). However, there might be a gender difference between HCV-associated and HBV-associated LC because the incidence and mortality of HCV-associated LC were higher in females but that of HBV-associated LC were higher in males (>70%).

Our study analyzed the proportion of five specific etiologies in LC incident cases. We showed that HBV infection remains the most important cause of LC in China, although the incidence of LC due to hepatitis B has been gradually decreasing (Supplementary Figure S3). The decrease in the proportion of HBV-associated LC could be explained by enormous efforts in China to control HBV infection over the past 30 years as well as the application of HBV treatments and vaccines (6). The incidence rates of five specific etiologies induced liver cancer have decreased over time. Since 1993, mandatory anti-HCV screening and other preventive measures to prevent the spread of blood-borne diseases have been widely implemented, therefore substantially reducing the spread of HCV in China (39). Furthermore, the drug discovery of direct-acting antiviral drugs (DAAs) has successfully cured many HCV-infected patients (40–45). In 2017, sofosbuvir was the first DAA approved by China Food and Drug Administration. Since then, the wide application of DAAs has gained popularity to treat HCV infections in China, whereas the absence of HCV vaccines hampers HCV prevention in China and other countries (46). During the past 30 years, China's alcohol consumption dramatically increased (47). This also made alcohol use the second most common cause of end-stage liver diseases. The easy access to cheap alcoholic drinks also challenges liver disease control in China (48). Moreover, the disease burden of LC caused by non-alcoholic steatohepatitis is increasing globally (20, 21), particularly in Asian countries (22). This is largely due to the improved economic conditions, urbanization, dietary and lifestyle changes, as well as the increased incidence of obesity and hyperlipidemia (17, 23, 24).

It is known that death-associated risk factors of liver cancer include marital status, race, gender, age (49, 50), alcohol and tobacco use (51), complications of diabetes, hypertriglyceridemia (52), and clinical characteristics such as grade and size of the tumor (53, 54). The comparative risk assessment from the GBD project has a risk-factor hierarchy that covers 4 levels of 87 risk factors. Risk factors at level 1 include behavioral, environmental, occupational, and metabolic factors. Three behavioral factors (tobacco use, alcohol use, and drug use) and one metabolic factor (high BMI) were selected to analyze their effect on liver cancer in our study. Our result showed that smoking (20%) was a leading factor associated with LC mortality. In China, the smoking population has barely declined over the past 30 years. Previous studies also suggested a higher risk of LC in smokers than in non-smokers (16, 55). Smoking is an independent risk factor for liver fibrosis, and 4,000 tobacco-related chemicals could cause a variety of body damage including liver damage (56, 57). Our study also revealed other risk factors, such as high BMI (10.1%), drug use (13.6%), and alcohol use (11.7%). Accumulated evidence suggests that overweight and obesity increase the risk of LC and deaths (58–60) and obesity is associated with an increasing burden of non-alcoholic steatohepatitis that causes liver cancer (61, 62). The association of alcohol with LC is known (63, 64) and alcohol is considered a liver toxin (65), which could increase the risk of adverse outcomes of liver diseases (66). Overall, the proportion of LC-associated deaths attributed to smoking, alcohol use, and high BMI was approximately 45%, with the other 55% attributed to drug use and other factors that are not covered in our study. Approximately 80% of liver cancer cases are associated with HBV/HCV infections, and the proportion of LC-associated deaths attributed to drug use was nearly 13.6%. It is known that drug use is associated with viral hepatitis (67–69) and effective control of drug use will help to reduce the incidence and mortality of LC.

We found a decline in the incidence, mortality, and DALY rate of liver cancer in China over the past 30 years, which is consistent with previous studies from other data resources (6, 34). This is probably due to the improvement of economic conditions, the increase in medical resources, and the development and application of new drugs. Based on the estimated population structure and LC data from 1990 to 2019, we used the Nordpred R package to predict LC incidence and mortality from 2020 to 2044. There will be a steady decrease in the incidence and mortality of liver cancer in both males and females, but both incidence and mortality rates are much higher in men than in women, suggesting that males will be the key group for prevention and control in the future.

Our study has limitations. First, the GBD 2019 database from the University of Washington (18, 19) is used in our study. Because the GBD 2019 dataset was estimated by the DisMod-MR 2.1 model (20), there might be some derivations but the database has been consistently maintained and corrected by the Institute for Health Metrics and Evaluation at the University of Washington. Second, our study included only four LC-associated factors, whereas it is known that LC incidence and mortality can be affected by many factors such as aflatoxin and the grade of the tumor. Third, treatment and prevention strategies exert an impact on the development of liver cancer. To reduce the disease burden of liver cancer in China, future studies are still needed to investigate LC-associated risk factors as well as effective treatment and prevention strategies.

## Conclusions

The disease burden of liver cancer in China has declined over the past 30 years. A high risk of liver cancer is commonly found among elderly males with high BMI, alcohol use, tobacco use, or drug abuse. Despite the decline in LC incidences and mortality in China, it remains a need to control liver cancer among high-risk populations with better treatment and prevention strategies.

## Data availability statement

The datasets presented in this study can be found in online repositories. The names of the repository/repositories and accession number(s) can be found below: http://ghdx.healthdata.org/gbd-results-tool.

## Ethics statement

Ethical review and approval was not required for the study because this study disclosed no personal information and used public data freely shared by the Global Burden of Disease (GBD) database (https://www.healthdata.org/about/data).

## Author contributions

TY performed statistical analyses and drafted the manuscript. MX and TC contributed with data interpretation. HZ, MP, and ED discussed the contents and ideas of the manuscript. GL obtained funding and revised the manuscript. All authors contributed to the article and approved the submitted version.

## Funding

This research was funded by the National Nature Science Foundation of China (31871324, 81730064, and 31571368), and the National Science and Technology Major Project (2018ZX10715004). The funders played no role in study design, data collection, data analysis, data interpretation, or writing of the report.

## Conflict of interest

The authors declare that the research was conducted in the absence of any commercial or financial relationships that could be construed as a potential conflict of interest.

## Publisher's note

All claims expressed in this article are solely those of the authors and do not necessarily represent those of their affiliated organizations, or those of the publisher, the editors and the reviewers. Any product that may be evaluated in this article, or claim that may be made by its manufacturer, is not guaranteed or endorsed by the publisher.

## Abbreviations

DALY: Disability-standardized life year
ASDR: Age-standardized DALY rate
ASIR: Age-standardized incidence rate
ASMR: Age-standardized mortality rate
APC: Annual percentage change
BMI: body mass index
GBD: the global burden of disease
LC: Liver cancer
CRA: Comparative Risk Assessment
DAAs: Direct-acting antiviral drugs.
