# Supplementary material for: Gender disparity and temporal trend of liver cancer in China from 1990 to 2019 and predictions in a 25-year period
Source: Front Public Health. 2022 Aug 26;10:956712. doi: 10.3389/fpubh.2022.956712 (PMC9459158; doi:10.3389/fpubh.2022.956712)
Supplement: Supplementary file 1 [file Data_Sheet_1.docx]

Supplementary Material

# Supplementary Figures

#
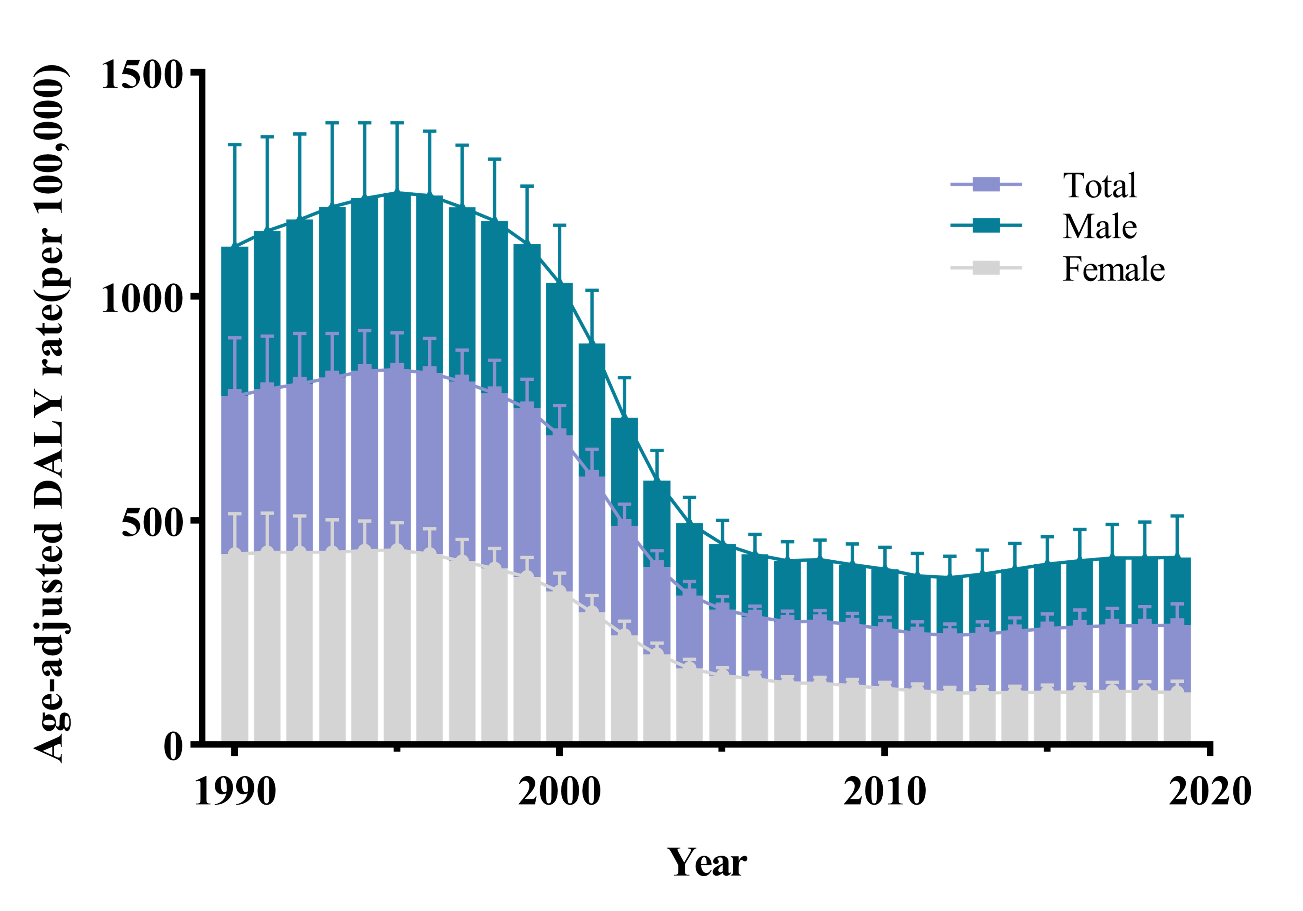


**Supplementary Figure 1.** Temporal trends of age-adjusted DALY rates for liver cancer from 1990 to 2019 in China.

#
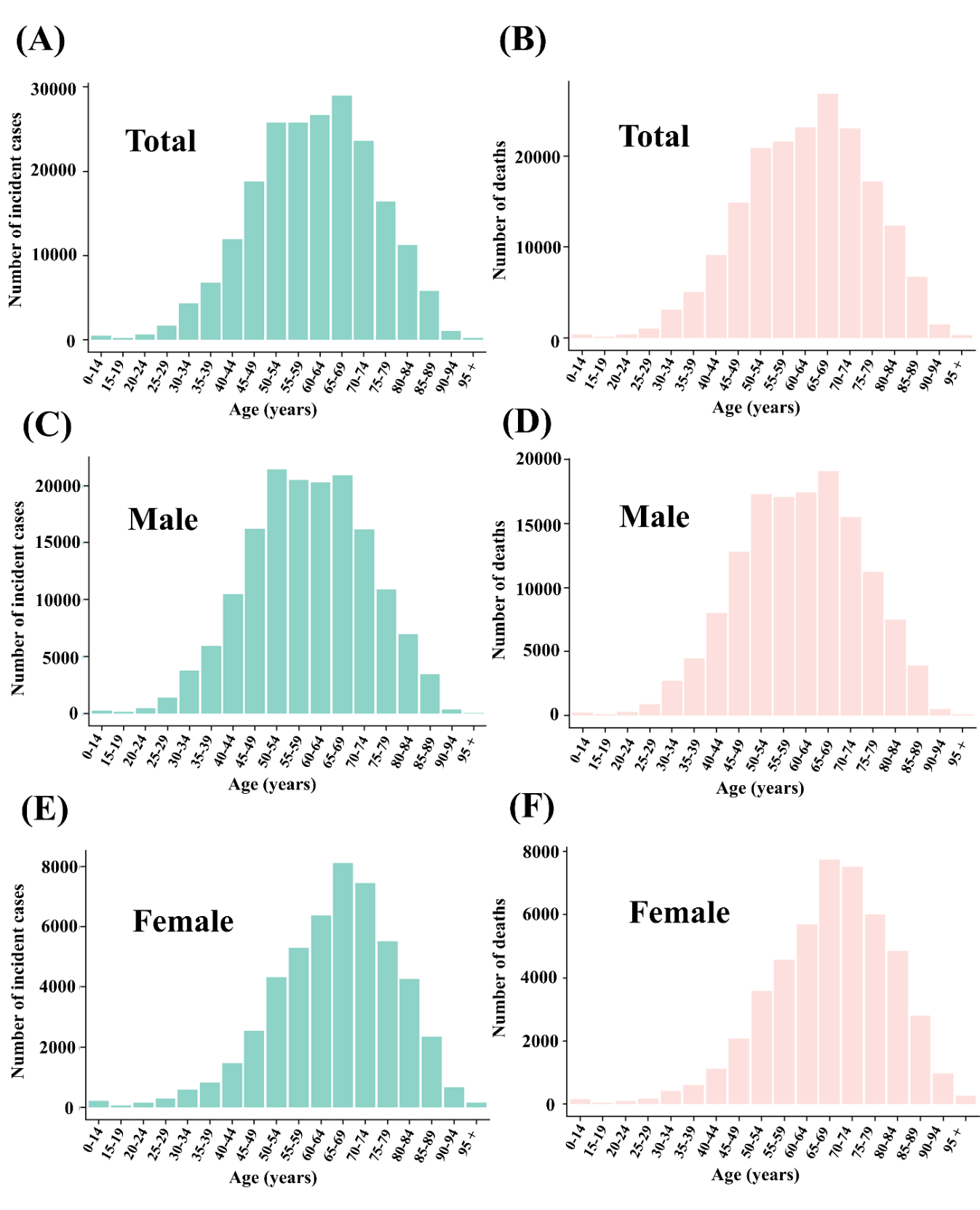


**Supplementary Figure 2.** Distribution of LC incident cases and Deaths in China. (A) Distribution of LC incident cases in different age groups; (B) Distribution of LC deaths in different age groups; (C) Age distribution of LC incident cases in males; (D) Age distribution of LC deaths in males; (E) Age distribution of LC incident cases in females; (F) Age distribution of LC deaths in females.


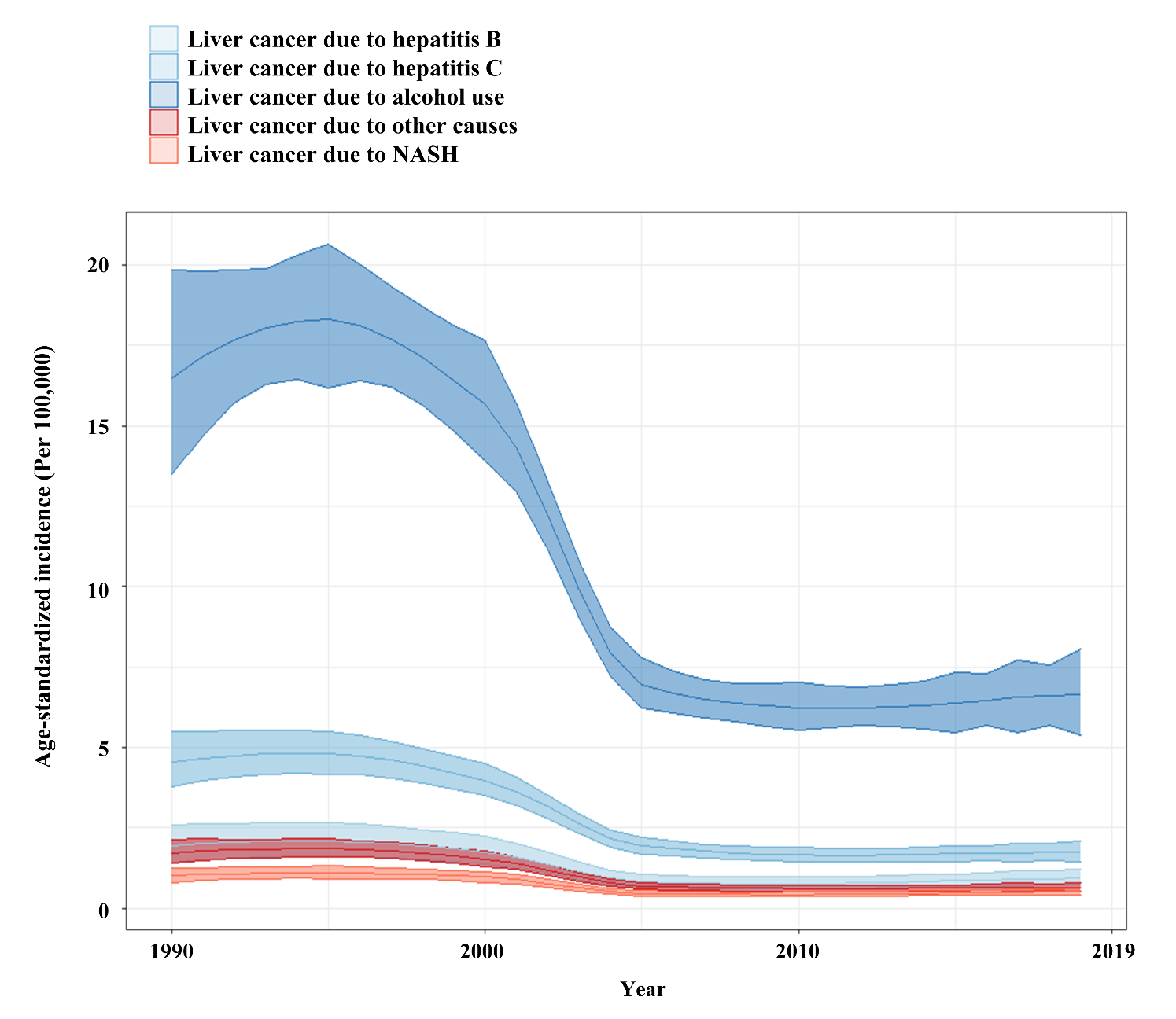


**Supplementary Figure 3.** Changing trend of age-standardized incidence of liver cancer in China, by specific etiologies, from 1990 to 2019.
